# Quality of life and functionality after total hip arthroplasty: a long-term follow-up study

**DOI:** 10.1186/1471-2474-12-222

**Published:** 2011-10-06

**Authors:** Massimo Mariconda, Olimpio Galasso, Giovan Giuseppe Costa, Pasquale Recano, Simone Cerbasi

**Affiliations:** 1 Department of Orthopaedic Surgery, Federico II University, Naples, Italy; 2 Department of Orthopaedic Surgery, Magna Graecia University, Catanzaro, Italy

## Abstract

**Background:**

There is a lack of data on the long-term outcome of total hip arthroplasty procedures, as assessed by validated tools.

**Methods:**

We conducted a follow-up study to evaluate the quality of life and functionality of 250 patients an average of 16 years (range: 11-23 years) after total hip arthroplasty using a validated assessment set including the SF-36 questionnaire, Harris Hip Score, WOMAC score, Functional Comorbidity Index, and a study specific questionnaire. Models of multiple stepwise linear and logistic regression analysis were constructed to evaluate the relationships between several explanatory variables and these functional outcomes.

**Results:**

The SF-36 physical indexes of these patients compared negatively with the normative values but positively with the results obtained in untreated subjects with severe hip osteoarthritis. Similar results were detected for the Harris Hip Score and WOMAC score. There was a 96% rate of post-surgical satisfaction. Hip functionality and comorbidities were the most important determinants of physical measures on the SF-36.

**Conclusions:**

Patients who had undergone total hip arthroplasty have impaired long-term self-reported physical quality of life and hip functionality but they still perform physically better than untreated patients with advanced hip osteoarthritis. However, the level of post-surgical satisfaction is high.

## Background

Hip osteoarthritis (OA) is a cause of severe pain and disability [1] but can be successfully treated with total hip arthroplasty (THA). Short- and medium-term THA studies report substantial improvements in the generic health-related quality of life (HRQoL) [2-6] and hip functionality [4,7] in subjects with OA. Currently about 20% of THA are performed in people younger than 60 years with variable diagnoses [8]; the general increase in life expectancy is expected to further increase the need for this procedure [9]. These data suggest that greater attention should be paid to the long-term follow-up results of hip replacement surgery. A comprehensive approach requires the combined use of generic and disease-specific patient-oriented validated measures [5], but there is a lack of data on the long-term outcome of THA procedures, as assessed by these validated tools. Even less is known about possible predictors of long term outcomes of these procedures. The goals of the present study were: 1) to evaluate by validated instruments whether subjects who had undergone THA more than 11 years earlier had severe functional impairment and/or disability, and 2) to identify possible outcome predictors of long term HRQoL and hip functionality after THA.

## Methods

**Figure 1:**
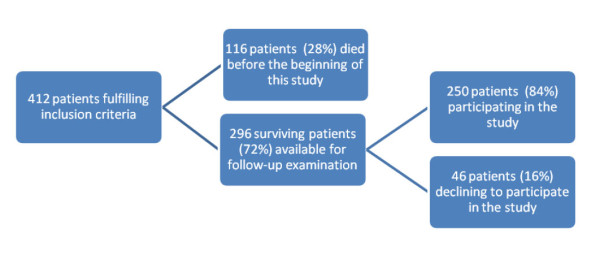
**Flow chart explaining the selection of patients**.

After approval by the local ethics committee, we enrolled patients who had undergone THA at our institution from 1985 to 1996 who fulfilled the following inclusion criteria: (1) age less than 70 years at operation (2) total hip arthroplasty, and (3) primary surgery. On the basis of these criteria we selected 412 subjects. One-hundred sixteen (28%) of them had died before our study commenced. Thus, 296 were available for follow-up examination. We were able to collect data on 250 patients (162 females and 88 males) with 330 THA (80 bilateral procedures), who represented 84% of the surviving patients. Forty-six subjects refused to participate in the study because of severe comorbidities or lack of interest. The selection of patients is shown in Figure 1. No significant differences were found between the participants and those subjects lost to follow-up with respect to gender (p = 0.83), preoperative diagnosis (p = 0.37), operating surgeon (p = 0.34), or use of cemented/cementless implant (p = 0.55). The only parameter that differentiated between the two groups was the mean age, which was significantly older in the subjects lost to follow-up (76.8 vs 70.8 years; p = 0.004). The patients' data are shown in Table 1. A direct transgluteal lateral approach was used in all cases. Out of 330 implants, 118 (36%) were cemented and 212 (64%) were cementless THA. Preoperative diagnoses were primary osteoarthritis in 252 hips (76%), osteonecrosis in 32 (10%), posttraumatic arthritis in 24 (7%), developmental dysplasia of the hip in 14 (4%), rheumatoid arthritis in 6 (2%), and residual arthritis from slipped capital femoral epiphysis in two hips (1%). The mean age at follow-up of eighty patients who received bilateral THA was older compared to subjects with unilateral THA (73 vs 69.7 years; p = 0.039), but no sex differences were found between these two groups. Out of 250 participants, 189 (76%) agreed to return for a follow-up visit, and 61 (24%) answered our questionnaires through a telephone interview. The mean ± standard deviation (SD) length of follow-up for the participants was 16.1 ± 3.6 years (range 11-23). During the follow-up visits, the patients gave their informed consent and underwent a complete physical examination as well as weight and height measurement. The clinical investigation was carried out by one of the authors, who was not involved in the primary care. The following patient-oriented instruments were chosen to evaluate the patients: the Italian version of the Short Form-36 Health Survey (SF-36) Questionnaire [10], the Harris Hip Score (HHS) [11], the Italian version of the Western Ontario and Mac Master University (WOMAC) Questionnaire [12], the Functional Comorbidity Index (FCI) [13], and a study-specific questionnaire dealing with patients' daily life activities, medical history, intensity and frequency of hip pain, possible reoperations, degree of satisfaction with surgery, and willingness to undergo the same operation again. The SF-36 Questionnaire is a generic measure of health status which contains 36 questions measuring the physical, social, and mental components of subjects. It yields an eight-scale profile of scores (i.e. physical functioning = PF; role physical = RP; bodily pain = BP; general health = GH; vitality = VT; social functioning = SF; role emotional = RE; mental health = MH) as well as summary physical (PCS) and mental (MCS) measures. SF-36 results were compared to the published data [14]. The HHS is a widely used disease-specific outcome measure for THA studies to assess pain and functional status. Sum scores are fitted in a 0-100 scale, with high values indicating less pain or better physical functioning. The WOMAC is a self-administered disease-specific validated outcome measure that evaluates pain (5 items), stiffness (2 items), and physical function (17 items). A total WOMAC summary score is calculated for each individual, adjusted, and reported on a 0-to-100 scale. Lower scores are associated with less pain and stiffness and better function. The FCI is a validated 18-item list of diagnoses designed to assess the burden of comorbidities on physical function. Each item is given 1 point if present, and the final score is the sum of the items. Fifteen randomly selected study subjects completed the questionnaires twice (the second time after a 20-day interval) to assess test-retest reliability. Pearson's product-moment correlation coefficients for the results of the tests ranged from 0.71 to 0.90 for the SF-36 scale scores, and averaged 0.84 and 0.86 for the HHS and WOMAC, respectively, and 0.90 for the FCI. The same outcome set was used for the participants who were interviewed by telephone. In these patients, since range of motion and deformity cannot be assessed by telephone, a modified HHS with a correction factor was adopted [15]. Since our study was carried out on surgically treated patients only without any control group including untreated patients, we compared our results with those obtained by other authors in patients affected by advanced hip osteoarthritis.

**Table 1:** Characteristics of the patients (n = 250)

Patients data	Mean ± SD (range) or N (%)
Age at the present follow-up	70.8 ± 11.8 (35-88)

Age at the operation	55.3 ± 11.7 (21-70)

Sex	
Female	162 (64.8%)
Male	88 (35.2%)

Body mass index	27.02 ± 4.3 (19-47.8)

Educational level	
Illiteracy	17 (6.8%)
Primary School	87 (34.8%)
Secondary School	78 (31.2%)
High School	55 (22%)
Graduation	13 (5.2%)

Cigarette smoking	
Non-smokers	178 (71.2%)
Ex-smokers	36 (14.4%)
Regular smokers	36 (14.4%)

Leisure activities	
None	232 (92.8%)
Sport	14 (5.6%)
Hobby	18 (7.2%)

Preoperative employment	
Employed	108 (43.2%)
Retired	32 (12.6%)
Housewife	110 (44.1%)

Change of job/workload after operation	
Yes	45 (18%)
No	205 (82%)

Change in strain at work after operation (subjects with change of postoperative employment)	
More sedentary	35 (77.8%)
Not different	10 (22.2%)

Functional Comorbidity Index	3.6 ± 1.9 (0-8)
0	14 (5.6%)
1	26 (10.4%)
2	28 (11.2%)
3	54 (21.6%)
4	50 (20%)
5	34 (13.6%)
6	24 (9.6%)
7	14 (5.6%)
8	6 (2.4%)

## Statistical analysis

A two-sample *t *test, ANOVA, and chi-square test were used to test the significance of the cross-sectional differences between groups. A Bonferroni test was used to test the differences between multiple groups. Pearson's correlation coefficient was used to assess the relationships among patient-oriented outcomes. Models of multiple stepwise linear and logistic regression analysis were constructed to evaluate the relationships between the explanatory variables and the outcomes with continuous and categorical distributions, respectively. Summary measures and single scale scores of the SF-36, as well as the WOMAC scores and the HHS, were treated as continuous outcome variables. Satisfaction with surgery, willingness to undergo the operation again, and occurrence of reoperation were categorical outcomes. Explanatory variables included in the analysis were: present age (continuous), gender (categorical), age at operation (continuous), bilaterality of the procedure (categorical), length of follow-up (continuous), BMI (continuous), educational level (discrete), FCI (discrete), cigarette smoking (categorical), sport practise (categorical), postoperative employment (i.e. keeping preoperative job/workload - categorical), cemented THA (categorical), and possible reoperations (categorical). The patients' educational level was graded as follows: 1) illiterate, 2) primary school, 3) secondary school, 4) high school, and 5) graduation. Before constructing the models, age-adjusted univariate linear and logistic regression analyses were performed. Explanatory variables were included in our multiple regression models if a trend toward an association (i.e. p ≤ 0.10) with the outcome of interest was found in the univariate analysis. In the multiple linear regression analysis, total R^2 ^for the model and changes in R^2 ^for the independent contribution of single factors were calculated to assess the percent of total variance in the outcome accounted for by the whole model and by single explanatory variables, respectively. In multiple logistic regression, log-likelihood tests were obtained to evaluate the independent contribution of single explanatory variables in the fit of the model. A *p-*value of less than 0.05 was considered significant. SPSS software program (SPSS, Inc., Chicago, IL, USA) was used for the database and statistics.

## Results

### General health

The subjects' SF-36 scores, stratified into three age groups, are reported in Table 2 in comparison with the age-matched normative data [14]. The SF-36 physical indexes of patients compared negatively with the normative values, mainly in the two youngest age groups. Significantly lower values were observed in the older age groups compared with the youngest age group. Patients with unilateral THA scored better than patients with bilateral THA on the RP (p < 0.001), GH (p < 0.05), SF (p < 0.05), and PCS (p < 0.05) SF-36 scales. The only difference between patients with different preoperative diagnoses were that better results were obtained by subjects with osteonecrosis compared to those with OA on the PCS (p = 0.022) scale. No significant differences were found between patients who received cementless or cemented THA, or who had undergone revision procedures or not during the follow-up period.

**Table 2:** SF-36 scores (mean ± SD) in patients (PTS) and age-matched population norms (CTR)[14]

			AGE GROUPS (years)			
	**≤ 64**		**65-74**		**≥ 75**	

**SF-36 scale**	**PTS (n = 50)**	CTR*	**PTS (n = 82)**	**CTR**	**PTS (n = 118)**	**CTR**

PF	58.4 ± 30^b,c^	87.5	44.1 ± 26	71.7 ± 24	37.4 ± 30	50.2 ± 33

RP	62.0 ± 37	79.7	50.0 ± 42	65.9 ± 38	54.7 ± 43	52.6 ± 50

BP	60.5 ± 26^a,d^	77.6	48.5 ± 20	67.6 ± 26	45.2 ± 19	53.3 ± 35

GH	50.6 ± 13^a,d^	65.0	41.2 ± 16	55.4 ± 19	35.9 ± 16	43.1 ± 25

VT	54.3 ± 13^b^	65.5	49.1 ± 16	59.3 ± 19	45.4 ± 17	46.7 ± 25

SF	66.0 ± 24^b^	79.4	57.6 ± 22	75.8 ± 23	52.7 ± 24	63.4 ± 30

RE	77.3 ± 35	77.0	65.3 ± 42	73.5 ± 34	67.4 ± 39	57.1 ± 48

MH	57.2 ± 10	70.1	54.5 ± 13	64.7 ± 19	55.9 ± 10	58.5 ± 25

PCS	40.9 ± 10^a,d^	49.5	35.3 ± 10	42.7 ± 9	32.5 ± 11	35.8 ± 11

MCS	45.0 ± 7	46.3	44.8 ± 9	45.8 ± 9	45.3 ± 7	41.4 ± 11

PF = Physical Functioning; RP = Role-Physical; BP = Bodily Pain; GH = General Health; VT = Vitality; SF = Social Functioning; RE = Role-Emotional; MH = Mental Health

*mean between 45-54 and 55-64 age decade norms

a P ≤ 0.001 vs. age group ≥ 75 years

b P ≤ 0.01 vs. age group ≥ 75 years

c P ≤ 0.05 vs. age group 65-74 years

d P ≤ 0.01 vs. age group 65-74 years

### Disease-specific quality of life

Mean HHS and WOMAC questionnaire results are reported in Table 3, stratified into three age groups in comparison with age-matched normative data when available [16]. Subjects from the study group obtained poorer scores in comparison with the values of healthy subjects, and the WOMAC function score was the most affected parameter. Patients aged 75 years or more had worst results when compared to the two younger age groups. No significant differences in HHS and WOMAC results emerged between patients operated on by different surgeons, with different diagnosis, with uni- or bilateral THA, or who underwent revision arthroplasty or not. The hip functionality was worse in subjects who changed their employment in the postoperative period, compared to those who did not (WOMAC score: p = 0.001; HHS: p < 0.01).

**Table 3:** HHS and WOMAC scores (mean ± SD) in patients (PTS) and age-matched population norms (CTR)[16]

	Age ≤ 64	Age 65 - 74		Age ≥ 75		All subjects	
	**PTS** **(N = 50)**	**PTS** **(N = 82)**	**CTR**	**PTS** **(N = 118)**	**CTR**	**PTS** **(N = 250)**	**CTR**

Total WOMAC	19.4 ± 20^a^	26.4 ± 17^b^	3.1 ± 6	34.5 ± 20	2.4 ± 3.8	28.8 ± 20	3.1 ± 7

Function	16.0 ± 16^a^	22.2 ± 14^c^	1.9 ± 4	28.1 ± 16	1.2 ± 2	23.6 ± 16	1.8 ± 5

Stiffness	1.1 ± 2	1.0 ± 2	0.3 ± 7	1.5 ± 2	0.4 ± 1	1.2 ± 2	0.4 ± 1

Pain	2.3 ± 4^a^	3.2 ± 3^b^	0.6 ± 1	4.9 ± 4	0.7 ± 1	3.8 ± 4	0.8 ± 2

Harris Hip Score	81.6 ± 17^a^	75.5 ± 14^c^	94.1 ± 10	68.8 ± 18	93.7 ± 7	74.8 ± 17	94 ± 82

a P ≤ 0.001 vs. age group ≥ 75 years

b P ≤ 0.01 vs. age group ≥ 75 years

c P ≤ 0.05 vs. age group ≥ 75 years

### Post-surgical satisfaction and revision rate

Of the 250 responders, 240 (96%) were satisfied with the outcome of their surgery and 242 subjects (96.8%) said that they would undergo the same procedure again. No difference in these outcomes was noted when patients operated on by different surgeons were compared. A preoperative diagnosis of hip dysplasia was associated with a lesser degree of postoperative satisfaction and willingness to undergo the surgery again. Indeed, the satisfaction rate was 97.5% and 66.6% in patients with such a diagnosis or not, respectively (p = 0.001). One-hundred eighty-two patients (72.8%) had experienced pain in their operated hip over time, which was referred to as mild and sporadic in 160 cases (87.9%) and moderate or continuous in 22 cases (12.1%). The intensity of pain on the 10-step visual analogic scale averaged 2.8 ± 2 (range 1-8). The pain appeared at exertion and/or in standing position in 174 (95.6%) and 78 (42.9%) patients, respectively. Seventy-two patients (28.8%) reported current consumption of pain alleviating medications. Forty-one THA in 36 patients were revised, leading to a reoperation rate of 12.4%. Five patients underwent a bilateral revision procedure. No difference in the reoperation rate was noted between the different operating surgeons or the preoperative diagnoses.

### Correlation and regression analysis

There were significant correlations between HHS and PCS (c = 0.69; p < 0.001), PCS and the WOMAC score (c = -0.71; p < 0.001), and HHS and the WOMAC score (c = -0.88; p < 0.001). A weaker but still significant correlation was noted between these three physical indexes and the MCS. Major determinants (i.e. those explaining a variation in the variance of the outcome of the model ≥ 3%) of the SF-36 summary and scale scores are reported in Table 4. The hip functionality assessed by either the WOMAC score or HHS (only the best predictor is reported in the table) was closely related to both physical and mental HRQoL. In our models, the variation in these indexes of hip functionality explained about half of the percent variance of PCS and PF scale scores. Comorbidities as assessed by FCI were another significant but less important determinant. A higher level of education showed a trend toward a positive association (p = 0.07) with some physical indexes on the SF-36 questionnaire (PCS, PF, and BP). Hip functionality (WOMAC score and HHS) (Table 5) was positively associated with the postoperative resumption of preoperative employment and negatively associated with age and with the number of comorbidities. The older the age at operation, the better the long-term WOMAC score, although this explanatory variable accounted for only a small amount of the variation in this disease-specific index. During our multivariate analysis, neither the bilaterality of the procedure, use of cemented or cementless implant, nor possible reoperations was found to be related to the WOMAC scores and HHS. The functionality of the operated hip (WOMAC score and HHS) was a major positive determinant of long-term satisfaction with the surgery and willingness to undergo the surgery again, whereas the number of comorbidities was negatively related to these outcomes. Results of the models including this outcome and its best functional predictor (the WOMAC score) are shown in Table 6. If the scores of the domains of the WOMAC scale were used separately as single determinants of postoperative satisfaction and willingness to undergo the surgery again, then the most relevant predictor was the pain scale score. Several factors were found to be associated with reoperation during the multivariate regression analysis (Table 6), but they had lesser importance as outcome predictors.

**Table 4:** Determinants of SF-36 scores; multiple linear regression analysis

			Outcome		
**Explanatory variable**	**c**	**95% CI**	***P***	Total adjusted R^2 ^%*	R^2 ^Change %**

			**PCS**		

WOMAC score	-0.28	-0.32 -- -0.22	< 0.001	64	49

FCI score	-1.83	-0.23 -- -1.35	< 0.001		12

			MCS		

HHS	0.16	0.11 -- 0.21	< 0.001	15	14

			PF		

WOMAC score	-0.92	-1.06 -- -0.79	< 0.001	60	54

FCI score	-3.53	-4.81 -- -2.25	< 0.001		5

			RP		

HHS	0.86	0.59 -- 1.14	< 0.001	24	16

Bilateral THA	-19,79	-29.72 -- -9.85	< 0.001		4

			BP		

HHS	0.60	0.49 -- 0.71	< 0.001	47	38

FCI score	-3.51	-4.60 -- -2.43	< 0.001		9

			GH		

HHS	0.38	0.30 -- 0.46	< 0.001		37

FCI score	-2.76	-3.56 -- -1.96	< 0.001	54	13

Follow-up length	-0.06	-0.09 -- -0.02	0.001		3

			VT		

WOMAC score	-0.30	-0.38 -- -0.21	< 0.001		34

FCI score	-2.87	-3.62 -- -2.12	< 0.001	50	13

Postoperative employment	5.52	2.07 -- 8.98	0.002		3

			SF		

HHS	0.65	0.53 -- 0.76	< 0.001	48	41

FCI score	-3.24	-4.34 -- -2.14	< 0.001		7

			RE		

HHS	0.97	0.73 -- 1.21	< 0.001	21	21

			MH		

HHS	0.27	0.20 -- 0.34	< 0.001	29	23

FCI score	-1.50	-2.19 -- -0.81	< 0.001		4

C = coefficient; CI = confidence interval; SF-36 = Short Form-36 Health Survey; PCS = physical component summary; MCS = mental component summary; PF = physical functioning; RP = role physical; BP = bodily pain; GH = general health; VT = vitality; SF = social functioning; RE = role emotional; MH = mental health; FCI = Functional Comorbidity Index

* = total adjusted R2 accounted for by the whole model

** = only explanatory variables accounting for a R2 variation in the outcome ≥ 3 are reported

**Table 5:** Determinants of WOMAC scores and HHS; multiple linear regression analysis

			Outcome		
**Explanatory variable**	**c**	**95% CI**	***P***	Total adjusted R^2 ^%*	R^2 ^Change %**

			**HHS**		

Age	-1.22	-1.77 -- -0.68	< 0.001		15
		
Postoperative employment	12.70	8.24 -- 17.16	< 0.001	35	11
		
FCI score	- 1.85	-2.92 -- -0.78	0.001		5

	Total WOMAC				

Postoperative employment	-14.89	-19.51 -- -10.27	< 0.001		13
		
FCI score	2.36	1.19 -- 3.52	< 0.001	30	10
		
Age	1.20	0.66 -- 1.75	< 0.001		4
		
Age at operation	-0.90	-1.43 -- -0.36	0.001		3

	WOMAC function				

FCI score	2.35	1.43 -- 3.28	< 0.001		13
		
Postoperative employment	-12.22	-15.90 -- -8.53	< 0.001	32	13
		
Age	0.92	0.49 -- 1.36	< 0.001		3
		
Age at operation	-0.71	-1.14 ---0.28	0.001		3

	WOMAC stiffness				

Postoperative employment	-0.70	-1.18 -- -0.22	0.004	5	3

	WOMAC pain				

Age	0.22	0.10 -- 0.33	< 0.001		8
		
Postoperative employment	-1.94	-2.90 -- -0.97	< 0.001	19	6
		
Age at operation	-0.14	-0.26 -- -0.03	0.017		3

C = coefficient; CI = confidence interval; FCI = Functional Comorbidity Index

* = total adjusted R2 accounted for by the whole model

** = only explanatory variables accounting for a R2 variation in the outcome ≥ 3 are reported

**Table 6:** Determinants of patients' satisfaction with surgery and reoperation; multivariate logistic regression analysis

Explanatory variables	OR	95% CI	*P*	- 2 Loglikelihood ratio (LR)
	Satisfaction			

WOMAC score	0.88	0.82-0.94	< 0.001	29.46

FCI score	0.37	0.16-0.86	0.021	9.95

	Willingness to undergo the operation again			

WOMAC score	0.83	0.73-0.95	0.007	23.59

FCI score	0.09	0.01-0.65	0.017	18.53

Age	1.12	1.00-1.24	0.041	5.01

	Reoperation			

Postoperative employment	0.34	0.15-0.78	0.011	6.33

Follow-up length	1.01	1.00-1.02	0.023	5.38

Cemented THA	0.38	0.15-0.99	0.048	4.34

OR = odds ratio; CI = confidence interval; WOMAC = Western Ontario and Mac Master University questionnaire; FCI = Functional Comorbidity Index; THA = total hip arthroplasty

## Discussion

The main result of the present study is that patients who had undergone THA a mean of 16 years earlier had poorer long-term HRQoL with respect to age-matched healthy controls [14]. However, their scores on physical SF-36 scales were higher in comparison with those previously reported in subjects with advanced hip osteoarthritis (Table 7) [1,17-20]. In the present study the older the age group, the lower the SF-36 scale scores and summary measures. To the best of our knowledge, no previous studies that used this validated instrument with a comparably long follow-up period have been published. Thus, making exact comparisons with our findings is impossible. Several prospective studies dealing with early results of THA have shown that patients may obtain normal age- and sex-adjusted SF-36 values 3-12 months after surgery [2,3,21], but twelve to 36 months after THA, SF-36 parameters start to decrease over time [20,22,23]. In the long term, Rat et al. [23] reported SF-36 scores similar to ours 10 years after THA. These authors also found that the scores on both physical and mental scales of SF-36 were lower than those for a general population with comparable age. Another long-term study [24] used a different validated questionnaire (i.e. the Nottingham Health Profile) that measures patient evaluation of the functional, social, and emotional impact of chronic disease. This study showed impaired quality of life in patients who had undergone THA 15 years earlier. These patients fared worse than the control group in most areas of perceived health. Moreover, they considered daily function to be affected negatively by health problems as compared with the control subjects. In our patients, also the indexes of hip functionality (the WOMAC questionnaire and HHS) compared negatively with those of healthy controls [16] but positively with those of patients with hip osteoarthritis [1]. Moreover, these scores were equal to or better than the findings of other THA studies with earlier follow-up data [22,25,26]. In our multivariate analyses, the WOMAC score and HHS were essential determinants of SF-36' PCS and PF scale scores, showing that hip functionality is critical in determining the patient's general functioning. In these models, comorbidities were negatively correlated with SF-36, WOMAC, and HHS results. This result is in keeping with previous studies that used the SF-36 [1,6,23] and WOMAC and HHS [1,21] questionnaires to evaluate either operated or non-operated subjects. The frequency of subjects who kept their preoperative employment after surgery was similar to other studies [27,28]. Resuming preoperative job or workload was closely associated with better hip functionality, as assessed by the WOMAC and HHS questionnaires. This is in good agreement with the results of Bohm [28], who found better Oxford-12 hip scores among those returning to work after THA. As stated by this author, a better hip functionality is likely to positively impact the ability to return to work, although this relationship may not be causal (i.e. the ability to resume work by itself may positively influence the patient's self reported functionality).

**Table 7:** SF-36 scores (mean ± SD) in the study group and in patients with advanced osteoarthritis

Author		Present study	Croft [17]	Nilsdotter [18]	Salaffi [1]*	Ng [19]	SooHoo [20]
Patients n.		250	611	124	107	627	89

Mean age (years)		70.8	70.9	71	67.8	68	60

SF-36 scale	PF	43.8 ± 30	28.9	31.5 ± 21	43.4 ± 23	23.1 ± 18	23
	
	RP	54.6 ± 41		11.5 ± 24	24.8 ± 35	6.6 ± 16	24
	
	BP	49.3 ± 22		31.2 ± 16	26.3 ± 21	27.4 ± 17	30
	
	GH	40.6 ± 17	56.2	69.7 ± 19	44.8 ± 22	74.3 ± 20	66
	
	VT	48.4 ± 17	60.4	48.7 ± 22	46.7 ± 20	43.9 ± 21	49
	
	SF	57.0 ± 24	48.7	66.6 ± 26	58.9 ± 24	53.7 ± 30	58
	
	RE	68.7 ± 39		40.0 ± 44	42.1 ± 32	45.2 ± 33	56
	
	MH	55.7 ± 11	64.5	69.7 ± 20	57.4 ± 22	71.8 ± 19	67
	
	PCS	35.1 ± 11			31.1 ± 8		31
	
	MCS	45.3 ± 8			38.9 ± 9		50

PF = Physical Functioning; RP = Role-Physical; BP = Bodily Pain; GH = General Health; VT = Vitality; SF = Social Functioning; RE = Role-Emotional; MH = Mental Health

*Patients not scheduled for THA with hip osteoarthritis of variable severity

Despite the impairment in the HRQoL, the level of post-surgical satisfaction in our study group was high and the 96% rate of satisfied patients is equal to or superior to the percentages previously reported in studies with shorter follow-up intervals [22,25,29]. This discrepancy between the rate of satisfied patients and HRQoL is not surprising. Indeed, several different factors apart from hip functionality (i.e. patient expectation, pain relief, psychological benefit, and improvement in activities of daily life) can influence the level of post-surgical satisfaction [30].

We acknowledge some methodological weaknesses in the present study. Due to its observational and retrospective character, it lacks reliable baseline data and a control group. However, performing a prospective analysis with such a follow-up is very challenging. Thus, we could have been subject to variability in information gathering that might have existed at the time these patients were treated. Nevertheless, the information obtained from medical records and used in the present analysis mostly consisted of unambiguous personal, demographic, or occupational data. Moreover, the comprehensive assessment by validated patient-oriented tools warranted comparisons with age and sex-matched norms, thus mitigating the lack of a control population. At the time the patients in our study group underwent their surgery, many of the validated questionnaires used in the present study were not available. This lack in comparable baseline data prevented us from evaluating the postoperative changes in these patients' status. However, this study was only designed to evaluate the influence of a THA on the long-term HRQoL and hip functionality of unselected patients. As stated in large register-based studies [31], the effectiveness of a widely used routine surgical technique (such as THA) can be evaluated better in observational studies than in randomised ones, because patients enrolled in these latter studies are frequently not representative of the entire cohort of subjects undergoing THA in the routine clinical practice, due to the stringent exclusion criteria. Lastly, although multiple attempts were made to trace all the patients for follow-up evaluation, this was impossible due to the long elapsed time and the death of many patients. Nevertheless, we obtained a satisfactory survey rate of more than 80% of the surviving patients, which is superior to other studies with shorter times until follow-up [6]. Comparison of the included patients with those lost to follow-up suggested that these participants were representative of the entire population. Main strengths of our study are the use of validated instruments and length of follow-up, since to the best of our knowledge, no previous studies that used these validated patient-oriented tools had similarly long intervals post-surgery.

## Conclusions

This paper demonstrates that patients who had undergone THA a mean of 16 years earlier have impaired self-reported physical HRQoL and hip functionality, but they still perform physically better than untreated patients with hip osteoarthritis. The hip functionality is a major determinant of physical HRQoL, but other relevant factors, such as the number of comorbidities, can also influence the ability of subjects. Despite the impairment in the HRQoL, the level of post-surgical satisfaction was high in this study group.

## Competing interests

The authors declare that they have no competing interests.

## Authors' contributions

MM and OG conceived and designed the study. GGC, PR, and SC gathered the data. MM, OG, GGC, PR, and SC analysed the data. MM, PR, and SC wrote the initial draft. MM, OG, and GGC checked the accuracy of data collection and analysis. MM and OG wrote the manuscript in its final form. All authors have given final approval to the manuscript.

## Pre-publication history

The pre-publication history for this paper can be accessed here:

http://www.biomedcentral.com/1471-2474/12/222/prepub
